# Safety of Ovaleap® (Follitropin Alfa) in Infertile Women Undergoing Superovulation for Assisted Reproductive Technologies: A Multinational Comparative, Prospective Cohort Study

**DOI:** 10.3389/fendo.2021.632674

**Published:** 2021-03-16

**Authors:** Sigal Kaplan, Rachel Levy-Toledano, Miranda Davies, Debabrata Roy, Colin M. Howles, Amir Lass

**Affiliations:** ^1^ Global Patient Safety & Pharmacovigilance, Teva Pharmaceutical Industries Ltd., Netanya, Israel; ^2^ RLT Media Consulting, Boulogne Billancourt, France; ^3^ Drug Safety Research Unit, Southampton, United Kingdom; ^4^ School of Pharmacy and Biomedical Sciences, University of Portsmouth, Portsmouth, United Kingdom; ^5^ Medical Department, Theramex Ltd., London, United Kingdom

**Keywords:** Ovaleap^®^, follitropin alfa, Gonal-f^®^, ovarian hyperstimulation syndrome, safety, pregnancy rate, live birth rate, pregnancy outcome

## Abstract

**Background:**

Ovaleap^®^ (follitropin alfa), a recombinant human follicle stimulating hormone, is a biosimilar medicinal product to Gonal-f^®^ and is used for ovarian stimulation. The main objective of this study was to assess the safety and effectiveness of Ovaleap^®^ compared to Gonal-f^®^ in one treatment cycle in routine clinical practice.

**Methods:**

Safety of Ovaleap^®^ Follitropin alfa in Infertile women undergoing superovulation for Assisted reproductive technologies (SOFIA) was a prospective cohort study conducted in six European countries. Eligible patients were infertile women undergoing superovulation for assisted reproductive technology, who were administered Ovaleap^®^ or Gonal-f^®^ for ovarian stimulation and were naïve to follicle stimulating hormone treatment. The recruitment ratio was 1:1. The primary endpoint was incidence proportion of ovarian hyperstimulation syndrome (OHSS) and the secondary endpoint was OHSS severity (Grades I, II, III). The effect of risk factors or potential confounders on the odds ratio for OHSS incidence as well as treatment effect on OHSS incidence was explored using univariate logistic regression. Pregnancy and live birth rates were also assessed.

**Results:**

A total of 408 women who were administered Ovaleap^®^ and 409 women who were administered Gonal-f^®^ were eligible for analysis. The incidence proportion of OHSS was 5.1% (95% CI: 3.4, 7.7) in the Ovaleap^®^ cohort and 3.2% (95% CI: 1.9, 5.4) in the Gonal-f^®^ cohort. This difference in OHSS incidence proportion between the two cohorts was not statistically significant neither before (p = 0.159) nor after univariate adjustment for each potential confounder (p > 0.05). The incidence proportion of OHSS severity grades was similar in the two treatment groups (3.4% versus 2.0% for Grade I, 1.2% versus 1.0% for Grade II, and 0.5% versus 0.2% for Grade III, in the Ovaleap^®^ and Gonal-f^®^ cohorts, respectively), without a significant statistical difference (p = 0.865, for each grade). Among patients who had embryo transfer, clinical pregnancy rates were 33% and 31% and live birth rates were 27% and 26%, in the two cohorts, respectively.

**Conclusions:**

Findings from the SOFIA study indicate that the incidence proportions of OHSS and OHSS severity, as well as pregnancy and live birth rates, are similar between Ovaleap^®^ and Gonal-f^®^ treatments and corroborate the safety and effectiveness of Ovaleap^®^ as a biosimilar to Gonal-f^®^.

## Introduction

A serious adverse outcome that may occur during assisted reproductive technology (ART) procedures is ovarian hyperstimulation syndrome (OHSS) (1, 2). OHSS is an iatrogenic complication caused by an excessive response to ovarian stimulation (3). It may manifest in various degrees of severity, from mild and moderate OHSS that resolve spontaneously, to severe OHSS requiring hospitalization (1). The incidence of moderate to severe OHSS has been estimated at 0.6% to 5% in ART cycles (4), and the incidence of milder forms may develop in up to 20%–30% of all *in vitro* fertilization (IVF) patients (5). Several risk factors for OHSS have been identified, including young age (<30 years), low body mass index (BMI), polycystic ovarian syndrome (PCOS), high basal antral follicle count, establishment of pregnancy during ART, human chorionic gonadotrophin (hCG) supplementation of the luteal phase, long menstrual cycle length, high basal serum anti-Mullerian hormone (AMH), and high or rapidly increasing serum estradiol and the use of a gonadotropin-releasing hormone (GnRH) agonist treatment cycle (3, 4). While a certain degree of ovarian hyperstimulation is expected with the use of follicle stimulating hormone (FSH), it is unclear when the symptoms evolve from the expected outcome to a disease state (5).

Ovaleap^®^ (Theramex, UK), a recombinant human FSH, is a biosimilar medicinal product to Gonal-f^®^ (Merck Europe Ltd.). A biosimilar medicine has to go through an exhaustive series of physico-chemical, *in vitro*, *in vivo* tests and confirmatory Phase I and Phase III studies, to demonstrate similarity/equivalence in quality, safety, and efficacy to the reference medicinal product, per the European Medicines Agency (EMA) guidelines (6). Ovaleap^®^ was approved by the EMA in 2013 for use at the same dose *via* the same route of administration and for the same therapeutic indications as Gonal-f^®^, including stimulation of multifollicular development in women undergoing superovulation for ART (7, 8).

Clinical comparability regarding efficacy and safety between Ovaleap^®^ and Gonal-f^®^ has been demonstrated in a randomized clinical trial carried out as part of the clinical development of the product (9, 10). During the trial, the incidence of OHSS was slightly higher, but without statistical significance, in the Ovaleap^®^ arm compared to the Gonal-f^®^ arm (4.6% versus 2.7%, respectively, p = 0.542). While randomized controlled studies are considered the gold standard for establishing treatment efficacy and generating evidence-based medicine, they have limited generalizability and are usually not powered to identify safety issues. Since Ovaleap^®^ is intended for use in a large population of women for stimulation of the ovaries, understanding the safety of Ovaleap^®^ in a real-world setting is important for patients, healthcare providers, and regulators. Such safety data are complementary to data derived from the clinical development program.

According to EMA guidelines, OHSS is an adverse reaction of special interest (6). As part of the risk management plan of Ovaleap^®^ in the European Union (EU), a post-authorization safety study (PASS) was required to assess the safety of Ovaleap^®^ among infertile women undergoing ART procedure in routine clinical practice (8). The primary objective of this study was to assess the safety of Ovaleap^®^ compared to Gonal-f^®^ during one treatment cycle with respect to the incidence proportion of OHSS in infertile women undergoing superovulation for ART. The secondary objective of the study was to examine the incidence proportion of OHSS severity grades [World Health Organization (WHO) Scientific Group classification (1973)] in Ovaleap^®^ compared to Gonal-f^®^. Pregnancy and live birth rates were also assessed.

## Material and Methods

### Study Design

SOFIA (**S**afety of **O**valeap^®^ **F**ollitropin alfa in **I**nfertile women undergoing superovulation for **A**ssisted reproductive technologies) was a multi-national, comparative, non-interventional, prospective cohort study. The study was performed at 56 centers specializing in ART in six European countries, Belgium, France, Germany, Italy, Spain, and the United Kingdom, from January 2017 to September 2019. The study was conducted in accordance with the approved protocol and local regulatory requirements. Ethics committee approval was obtained in all participating countries. All participants provided their written informed consent to participate in this study. SOFIA was registered on the EU electronic Register of Post-Authorization Studies (EUPAS17328).

### Study Participants

The study population comprised of infertile women undergoing superovulation for ART, and who were administered Ovaleap^®^ or Gonal-f^®^ for ovarian stimulation, and were naïve to any FSH product (i.e., recombinant or urinary-derived) or any product containing FSH activity (i.e., human menopausal gonadotropin [hMG]). Women were excluded if they had one of the following conditions: (a) primary ovarian failure; (b) ovarian enlargement or cyst; (c) reproductive system neoplasm; (d) prior history of OHSS; (e) known allergy or hypersensitivity to recombinant FSH preparations; (f) gynecologic bleeding; or (g) contraindications to receive recombinant human FSH. Given that these pre-existing conditions are contraindicated with the use of FSH products, exclusion of these women eliminated any potential effect of these conditions on the study outcomes.

Women were considered for enrollment in the study after the participating physicians decided on the treatment regimen according to their routine clinical practices. Eligible patients were enrolled at a ratio of approximately 1:1, both within and between countries. According to the Summary of Product Characteristics, patients self-administered daily subcutaneous injections of Ovaleap^®^ or Gonal-f^®^ (7). They were followed for one treatment cycle (of up to 20 days) as part of their routine medical care between first administration of Ovaleap^®^ or Gonal-f^®^ and up to 30 days after the last dose administration, for a total follow-up of up to 50 days. In addition, women who had fresh embryo transfer and a confirmed clinical pregnancy were followed until the end of the pregnancy or until delivery.

### Measurements

During study entry and follow-up period, data were collected from the centers based on patient’ medical records. Demographics, baseline characteristics, exposure to Ovaleap^®^ and Gonal-f^®^, comorbidities, concomitant medications, reproductive history, potential confounding factors (relevant measurements performed around the time of the IVF treatment administration cycle), biochemical pregnancy and pregnancy outcome, where applicable, were recorded in specific case report forms for each cohort member. A summary of the information planned to be collected in the routine visits expected during the IVF cycle is provided in **Supplementary Table 1**. Comorbidities were encoded using the Medical Dictionary for Regulatory Activities (MedDRA, version 19.1). Concomitant medications were encoded according to the WHO drug dictionary (WHODrug) and Anatomical Therapeutic Chemical (ATC) classification system, as appropriate.

### Exposure

Detailed information on the study drug administration was collected including dates of administration, dose, and duration of treatment. In addition, data were collected on the use of GnRH antagonist or GnRH agonist for pituitary desensitization, the use of hCG or GnRH agonists (type, dose, time of administration) for oocyte maturation, medications used in the luteal phase support, and concomitant medications (indication, route of administration, dose, frequency, start and stop dates).

### Study Endpoints

The primary endpoint was incidence proportion of OHSS which was initially identified according to patient symptoms and subsequently validated by physician’s diagnosis and medical records. The secondary endpoint was the severity grade of OHSS cases classified according to the WHO Scientific Group criteria (11) as follows: (a) Grade I (mild) - characterized by ovarian enlargement (ovary size 5 to 7 cm), may be accompanied by abdominal discomfort of varying degrees; (b) Grade II (moderate) - characterized by distinct ovarian cysts (ovary size 8 to 10 cm), accompanied by abdominal pain and tension, nausea, vomiting, and diarrhea; and (c) Grade III (severe) - characterized by enlarged cystic ovaries (ovary size >10 cm), accompanied by ascites and occasionally hydrothorax. In rare cases, Grade III OHSS may be further complicated by the occurrence of thromboembolic events.

Clinical pregnancy was classified as positive in participants who had an ultrasound with a detectable fetal heartbeat. Live birth was defined as deliveries that resulted in a live born neonate.

### Statistical Methods

Based on clinical trial data, the incidence proportion of OHSS was reported to be 3.6% (8). Assuming an OHSS incidence proportion of 4% in both cohorts and a sample size of 410 patients per cohort, the upper limit of the observed one-sided 97.5% confidence interval (CI) for the difference in OHSS incidence proportion between Ovaleap^®^ and Gonal-f^®^ was expected to be less than 4% with 80% power.

The full analysis set included all enrolled patients who received at least one dose of Ovaleap^®^ or Gonal-f^®^. Patients who received additional FSH products while being treated with Ovaleap^®^ or Gonal-f^®^ were excluded from the full analysis set. A patient that switched between the two treatments prior to receiving the first dose was analyzed according to the treatment received.

Descriptive statistics for patients’ characteristics and demographics, drug treatment, and diagnoses are provided for the study population, where available. Categorical variables are presented as counts (n), and percentages (%). Continuous variables are presented as means with standard deviation (SD), and medians with minimums and maximums.

The incidence proportion of OHSS (with 95% CIs) following IVF treatment was estimated in each cohort. The incidence proportion difference with 95% CIs was calculated using the Newcombe-Wilson method (12). The chi-squared test or Fisher’s exact test was used to estimate p-values for the difference in incidence proportions between the two treatments. The effect of risk factors or potential confounders on the odds ratio for OHSS incidence as well as treatment effect on OHSS incidence was explored using univariate logistic regression. In addition, the severity grades of OHSS by treatment group was assessed.

Clinical pregnancy rates were calculated per number of patients who had embryo transfer. In addition, pregnancy outcomes including live birth rates and major congenital anomalies were assessed. All data analyses were performed using SAS software version 9.4 (SAS Institute Inc., Cary, NC, USA).

## Results

### Study Population and Baseline Characteristics

**Figure 1:**
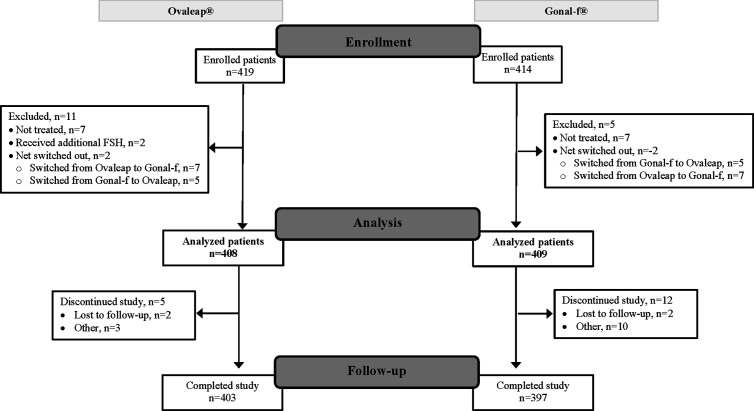
Flow diagram of patient disposition: from enrollment to analysis and study completion. FSH, follicle stimulating hormone.

A total of 817 enrolled patients were included in the full analysis set, 408 patients in the Ovaleap^®^ cohort and 409 patients in the Gonal-f^®^ cohort (**Figure 1**). Of these, 800 patients completed the study, including 403 patients (99%) in the Ovaleap^®^ cohort and 397 patients (97%) in the Gonal-f^®^ cohort. A total of 772 patients completed FSH treatments (i.e., up to oocytes maturation triggering), including 382 patients (94%) in the Ovaleap^®^ cohort and 390 patients (95%) in the Gonal-f^®^ cohort. The two cohorts were generally similar with regard to demographic and baseline characteristics. Overall, the mean age of women receiving FSH was 34 years (SD = 4.6) and ranged from 19 to 45 years (**Table 1**). More than 40% of the patients in each cohort were aged >34 years and the percentage of women younger than 30 years was slightly higher in the Ovaleap^®^ cohort. Overall, the majority of patients were Caucasian origin (85%) and had a BMI over 18.5 kg/m^2^ (>90%). The frequency of PCOS, high antral follicle count (≥12) and high basal serum level of AMH (≥3.5 ng/ml) was slightly higher in the Ovaleap^®^ compared to the Gonal-f^®^ cohort: 5% versus 3% for PCOS; 12% versus 10% for antral follicle count of right ovaries; 16% versus 10% for antral follicle count of left ovaries; and 27% versus 25% for basal serum AMH level, respectively.

**Table 1:** Demographics and baseline characteristics at initiated cycle for Ovaleap**^®^** and Gonal-f**^®^** cohorts, SOFIA study.

Characteristics	Ovaleap^®^	Gonal-f^®^	Total
	N = 408	N = 409	N = 817
**Age (years)**			
n	408	409	817
Mean (SD)	34 (4.7)	34 (4.6)	34 (4.6)
Median (min, max)	34 (20, 44)	35 (19, 45)	34 (19, 45)
**Age group (years), n (%)**			
18–29	85 (21)	62 (15)	147 (18)
30–34	154 (38)	134 (33)	288 (35)
≥35	169 (41)	213 (52)	382 (47)
**Ethnic origin, n (%)**			
Caucasian	339 (83)	352 (86)	691 (85)
Black	14 (3)	9 (2)	23 (3)
Asian	16 (4)	11 (3)	27 (3)
Hispanic	5 (1)	4 (1)	9 (1)
Other	8 (2)	13 (3)	21 (3)
Missing	26 (6)	20 (5)	46 (6)
**Country**			
Spain	99 (24)	110 (27)	209 (26)
Italy	101 (25)	107 (26)	208 (25)
Germany	77 (19)	86 (21)	163 (20)
France	80 (20)	61 (15)	141 (17)
United Kingdom	29 (7)	21 (5)	50 (6)
Belgium	22 (5)	24 (6)	46 (6)
**BMI (kg/m^2^) group, n (%)**			
<18.5	18 (4)	25 (6)	43 (5)
18.5 to <25	257 (63)	240 (59)	497 (61)
≥25	132 (32)	142 (35)	274 (34)
Missing	1 (<1)	2 (<1)	3 (1)
**Current smoker, n (%)**			
Yes	76 (19)	73 (18)	149 (18)
No	330 (81)	331 (81)	661 (81)
Unknown	2 (<1)	5 (1)	7 (1)
**Previous pregnancies, n (%)**			
No	256 (63)	264 (65)	520 (64)
Yes	152 (37)	145 (35)	297 (36)
**PCOS, n (%)**			
No	386 (95)	398 (97)	784 (96)
Yes	22 (5)	11 (3)	33 (4)
**Antral follicle count − right ovary, n (%)**			
<12	256 (63)	268 (66)	524 (64)
≥12	49 (12)	39 (10)	88 (11)
Missing	103 (25)	102 (25)	205 (25)
**Antral follicle count − left ovary, n (%)**			
<12	243 (60)	266 (65)	509 (62)
≥12	64 (16)	41 (10)	105 (13)
Missing	101 (25)	102 (25)	203 (25)
**Basal serum level of AMH (ng/ml), n (%)**			
<3.5	199 (49)	216 (53)	415 (51)
≥3.5	112 (27)	102 (25)	214 (26)
Missing	97 (24)	91 (22)	188 (23)

AMH, anti-Muellerian hormone; BMI, body mass index; min, minimum; max, maximum; n, number; PCOS, polycystic ovary syndrome; SD, standard deviation.

N was used as the denominator for calculating the percentages.

Percentage may not add up to 100% because of rounding.

### IVF Treatment

GnRH antagonist was the most frequent protocol used for ovarian stimulation treatment (84%) in both cohorts (**Table 2**). The mean duration of FSH treatment in the Ovaleap^®^ and Gonal-f^®^ cohorts was 10 days (SD were 2.0 and 1.9, respectively). Only one fifth of patients had reduction in FSH dose during stimulation in both Ovaleap^®^ and Gonal-f^®^ cohorts. The mean total doses received were 2,065 international units (IU) (SD = 805.4) and 2040 IU (SD = 855.1), respectively. Oocyte maturation triggering was performed in 91% and 95% of patients treated with Ovaleap^®^ and Gonal-f^®^, respectively. The primary medication used for triggering was hCG (84% and 85%, respectively). The most frequent reason for not performing oocyte maturation triggering was insufficient response to FSH treatment. Over-response to FSH treatment (to prevent OHSS) or cycle cancellation due to OHSS were not reported. Oocyte retrieval was performed in the majority of patients in both cohorts (>90%). The median numbers of oocytes retrieved were 10 (range: 0 to 43) and 8 (range: 0 to 29) in the Ovaleap^®^ and Gonal-f ^®^ cohorts, respectively. Medications commonly used in the luteal phase support were progesterone preparations or progestins (69% and 75% in the Ovaleap^®^ and Gonal-f^®^ cohorts, respectively).

**Table 2:** Protocol used during *in vitro* fertilization for Ovaleap**^®^** and Gonal-f**^®^** cohorts, SOFIA study.

Characteristics	Ovaleap^®^	Gonal-f^®^	Total
	N = 408	N = 409	N = 817
**Ovarian stimulation protocol, n (%)**			
GnRH agonist	53 (13)	52 (13)	105 (13)
GnRH antagonist	342 (84)	344 (84)	686 (84)
Missing	13 (3)	13 (3)	26 (3)
**Duration of treatment** ^a^ (days)			
Mean (SD)	10 (2.0)	10 (1.9)	N/A
Median (min, max)	10 (4, 16)	10 (5, 19)	N/A
**FSH dose reduction**			
No	327 (80)	337 (82)	664 (81)
Yes	81 (20)	72 (18)	153 (19)
**Total dose received (IU)**			
Mean (SD)	2,065 (805.4)	2,040 (855.1)	N/A
Median (min, max)	1,875 (750, 5,400)	1,925 (600, 6,750)	N/A
**Oocyte maturation triggering, n (%)**			
No	36 (9)	21 (5)	57 (7)
Yes	372 (91)	388 (95)	760 (93)
hCG^b^	313 (84)	331 (85)	644 (85)
GnRH agonist^b^	56 (15)	51 (13)	107 (14)
Missing^b^	3 (1)	6 (2)	9 (1)
**Oocyte retrieval, n (%)**			
Yes	370 (91)	382 (93)	752 (92)
**Number of oocytes retrieved**			
Mean (SD)	11 (6.8)	9 (6.1)	10 (6.5)
Median (min, max)	10 (0, 43)	8 (0, 29)	9 (0, 43)
**Medications used in the luteal phase support**^**c**^**, n (%)**			
Progesterone or progestin	284 (70)	305 (75)	589 (72)
GnRH analog	19 (5)	8 (2)	27 (3)
hCG	8 (2)	10 (2)	18 (2)
Missing	133 (33)	110 (27)	243 (30)

FSH, follicular stimulating hormone; GnRH, gonadotropin-releasing hormone; hCG, human chorionic gonadotropin; IU, international units; max, maximum; min, minimum; n, number; N/A, not applicable; SD, standard deviation.

a Total duration was calculated as last day of study drug – first day of study drug + 1.

b Number of patients with oocyte maturation triggering was used as the denominator for calculating the percentages.

c More than one medication may have been used.

Unless otherwise noted, N was used as the denominator for calculating the percentages.

### OHSS

The incidence proportion of OHSS was 5.1% (95% CI: 3.4, 7.7) in the Ovaleap^®^ cohort and 3.2% (95% CI: 1.9, 5.4) in the Gonal-f^®^ cohort (**Table 3**). The difference in incidence proportion of OHSS between the Ovaleap^®^ and Gonal-f^®^ cohorts was 1.9% (95% CI: −0.8, 4.9) and was not statistically different (p = 0.159). OHSS incidence proportions stratified by risk factors or potential confounders and treatment group are summarized in **Supplementary Table 2**.

**Table 3:** Incidence proportion of ovarian hyperstimulation syndrome for Ovaleap**^®^** and Gonal-f**^®^** cohorts, SOFIA study.

	Patients treated (at risk)	OHSS cases	Incidence proportion	95% CI^a^	Incidence proportion difference	95% CI^a^	p-value
	N	n	n/N (%)		%		
**Ovaleap^®^**	408	21	5.1	3.4–7.7	1.9	−0.8–4.9	0.159
**Gonal-f^®^**	409	13	3.2	1.9–5.4			

CI, confidence interval; OHSS, ovarian hyperstimulation syndrome.

a CIs were estimated using the Newcombe-Wilson score method. The p-value was based on the chi-squared test or Fisher’s exact test if there were fewer than five events in any category.

In a univariate regression model, risk factors and potential confounders such as PCOS, embryo transfer, antral follicle count, basal serum level of AMH, pregnancy, FSH dose reduction, and FSH treatment duration were significantly associated with OHSS incidence (p < 0.05) (**Supplementary Table 3**). However, no statistically significant difference in OHSS incidence was observed in the odds ratio for treatment effect between the Ovaleap^®^ and Gonal-f^®^ cohorts after adjusting for each risk factor or potential confounder (p > 0.05).

The incidence proportions of OHSS severity grades were 3.4% versus 2.0% for Grade I, 1.2% versus 1.0% for Grade II, and 0.5% versus 0.2% for Grade III, for the Ovaleap^®^ cohort compared to Gonal-f^®^ cohort, respectively, and differences were not statistically significant (p = 0.865, for each grade) (**Table 4**). Overall, the majority of patients with OHSS in the Ovaleap^®^ and Gonal-f^®^ cohorts were at Grade I (mild) (67% and 62%, respectively) or Grade II (moderate) (24% and 31%, respectively) levels.

**Table 4:** Severity grades of ovarian hyperstimulation syndrome according to the World Health Organization (WHO) Scientific Group criteria for Ovaleap**^®^** and Gonal-f**^®^**, SOFIA study.

OHSS severity grade	Ovaleap^®^	Gonal-f^®^	P-Value
	N = 408	N = 409	
	n (%)	n (%)	
**Total patients with OHSS**	21 (5.1)	13 (3.2)	
Grade I (mild)	14 (3.4)	8 (2.0)	0.865
Grade II (moderate)	5 (1.2)	4 (1.0)	0.865
Grade III (severe)	2 (0.5)	1 (0.2)	0.865

n, number; OHSS, ovarian hyperstimulation syndrome.

N was used as the denominator for calculating the percentages.

### Embryo Transfer and Pregnancy Outcomes

Approximately two-thirds of patients underwent fresh embryo transfer, including 256 patients (63%) in the Ovaleap^®^ cohort and 274 patients (67%) in the Gonal-f^®^ cohort (**Table 5**). Among patients who did not have embryo transfer performed, the most common reasons for not performing embryo transfer in the Ovaleap^®^ and Gonal –f^®^ cohorts, respectively, were: no embryo obtained (28% and 32%), “freeze-all” procedures (21% and 27%), high risk of OHSS (20% and 20%), no oocytes or oocytes retrieval not performed (16% and 9%). Single embryo transfer was performed in the majority of patients (56% and 58% in the Ovaleap^®^ and Gonal-f^®^ cohorts, respectively). The mean number of fresh embryos transferred was 1.5 (SD = 0.56) and 1.5 (SD = 0.57) in the Ovaleap^®^ and Gonal-f^®^ cohorts, respectively. Three-quarters of the total number of transfers were performed using late stage (4 to >5 day) cleavage embryos with a similar frequency in both cohorts.

**Table 5:** Embryo transfer practices, pregnancy and fetal outcomes for Ovaleap**^®^** and Gonal-f**^®^** cohorts, SOFIA study.

Outcome	Ovaleap^®^	Gonal-f^®^	Total
	N = 408	N = 409	N = 817
**Fresh embryo transfer, n (%)**			
Yes	256 (63)	274 (67)	530 (65)
No	136 (33)	116 (28)	252 (31)
Missing	16 (4)	19 (5)	35 (4)
**Reason for not performing fresh embryo transfer, n (%)**	136 (100)	116 (100)	252 (100)
No embryos obtained	38 (28)	37 (32)	75 (30)
All embryos were frozen (Freeze all)	29 (21)	31 (27)	60 (24)
High risk of OHSS	27 (20)	23 (20)	52 (21)
No oocytes or oocytes retrieval not performed	22 (16)	10 (9)	32 (13)
Ongoing OHSS	7 (5)	7 (6)	12 (5)
Other reason	13 (10)	8 (7)	21 (8)
**Number of embryos transferred, n (%)**			
Number of patients with embryos transferred	256 (100)	274 (100)	530 (100)
1 embryo transferred	143 (56)	159 (58)	302 (57)
>1 embryo transferred	113 (44)	115 (42)	228 (43)
**Embryos transfer**			
Mean (SD)	1.5 (0.56)	1.5 (0.57)	1.5 (0.57)
Median (min, max)	1 (1, 3)	1 (1, 3)	1 (1, 3)
**Time between oocyte retrieval and embryo transfer** ^a^ **, n (%)**			
N	256	274	530
<2 days	2 (<1)	3 (1)	5 (1)
2 to 3 days	63 (25)	66 (24)	129 (24)
4 to 5 days	97 (38)	104 (38)	201 (38)
>5 days	94 (37)	101 (37)	195 (37)
**Clinical pregnancy** ^b^			
Number of clinical pregnancy	84	86	170
Rate per embryo transfer^c^, n/N (%)	84/256 (33)	86/274 (31)	170/530 (32)
**Live births**	68	71	139
Rate per embryo transfer, n/N (%)	68/256 (27)	71/274 (26)	139/530 (26)
**Spontaneous abortions**			
Rate per clinical pregnancy, n/N (%)	12/84 (14)	10/86 (12)	22/170 (13)
**Elective terminations**			
Number of elective terminations	2	1	3
Rate per clinical pregnancy, n/N (%)	2/84 (2)	1/86 (1)	3/170 (2)
**Intrauterine deaths**			
Number of intrauterine deaths	1	0	1
Rate per clinical pregnancy, n/N (%)	1/84 (1)	0	1/170 (1)
**Twins**			
Number of twins	5	9	14
Rate per live birth, n/N (%)	5/68 (7)	9/71 (13)	14/139 (10)
**Major congenital anomaly**			
Number of congenital anomalies	1	2	3
Rate per live birth, n/N (%)	1/68 (1)	2/71 (3)	3/139 (2)

n, number; N, total number within a category.

The denominator used for calculating the percentage was the number in each category.

a Calculated as: date of transfer - date of oocyte retrieval + 1.

b Based on sonographic diagnosis.

c Among patients who had embryo transfer. The denominator in the Gonal-f^®^ cohort includes 4 patients with missing information on clinical pregnancy.

Among patients who had embryo transfer, clinical pregnancy rates were 33% and 31% in the Ovaleap^®^ and Gonal-f^®^ cohorts, respectively. The live birth rates per embryo transfer were 27% and 26% in the Ovaleap^®^ and Gonal-f^®^ cohorts, respectively.

Spontaneous abortion rates per clinical pregnancy were 14% and 12%, respectively. The frequencies of twin births per live birth were 7% and 13%, respectively. Major congenital anomalies were reported in one live birth infant in the Ovaleap^®^ cohort and two live birth infants in the Gonal-f^®^ cohort.

## Discussion

Findings from SOFIA study, a large multi-national prospective cohort study that used primary data collection, indicate that the safety and effectiveness profile of Ovaleap^®^ is similar to that of Gonal-f^®^ with regards to the incidence proportion of OHSS, ovarian stimulation characteristics and the rates of pregnancy and live birth in infertile women undergoing superovulation for ART in routine clinical practice. The incidence proportions of OHSS in Ovaleap^®^ and Gonal-f^®^ cohorts (5.1% and 3.2%, respectively) were not significantly different (p = 0.159) and were within the range of OHSS incidence proportions found in the literature (4, 5). The small difference in OHSS incidence proportions between Ovaleap^®^ and Gonal-f^®^ cohorts, while not associated with FSH treatment, may reflect subtle differences in the baseline characteristics between the cohorts. Specifically, compared to Gonal-f^®^, the Ovaleap^®^ cohort included a higher frequency of women younger than 30 years of age, of PCOS, of antral follicle count ≥12 and of higher basal serum level of AMH. Adjustment for each risk factor or potential confounder in the univariate logistic regression analysis further corroborated the finding of no difference in OHSS incidence between Ovaleap^®^ and Gonal-f^®^ treatments. These results are consistent with those reported in the clinical development program of Ovaleap^®^ and in a post marketing study (9, 10, 13). Moreover, little difference was observed between the cohorts within each severity grading and OHSS severity level incidence proportions were compatible with the background rate described in the literature (4, 5).

Previously identified OHSS risk factors (3, 4) that were confirmed to be associated with the incidence of OHSS in the SOFIA study include PCOS, having an embryo transfer, antral follicle count, basal serum AMH, FSH dose reduction, FSH treatment duration, and pregnancy. Other risk factors such as age and BMI were not found to be associated with OHSS, possibly due to the fact that a minority of the women were younger (<30 years) and/or had a low BMI. Overall, an appropriate representation of women with clinical characteristics compatible with high OHSS risk were included, further emphasizing the relevance of these results to routine clinical practice.

In line with evolving IVF clinical practice, the majority of patients in the two treatment groups (84% in each) received a GnRH antagonist as part of the ovarian stimulation protocol. The use of a GnRH antagonist as the primary pituitary desensitizing agent during ovarian stimulation has enabled more appropriate treatment segmentation to drastically reduce the risk of OHSS (14–16).

The findings that OHSS incidence proportion as well as ovarian simulation characteristics, clinical pregnancy and live birth outcomes do not differ between Ovaleap^®^ and Gonal-f^®^ is to be expected, given that Ovaleap^®^ is a biosimilar to Gonal-f^®^, with both containing follitropin alfa. As a biosimilar, Ovaleap^®^ meets the EMA’s clinical requirements for recombinant human FSH-containing medicinal products by demonstrating comparability to the already marketed reference product, Gonal-f^®^ (6). Other biosimilars for Gonal-f^®^ have been developed and examined in clinical trials, and have been supported by their similar therapeutic and safety profiles, including their similar OHSS incidence proportions (17–19). The clinical efficacy of Ovaleap^®^ has been examined in recently published studies that demonstrated similar ongoing clinical pregnancy or cumulative live birth rates compared to other follitropin alfa and follitropin beta preparations (20, 21).

Recently a meta-analysis was performed based on data derived from clinical trials of two biosimilar preparations (Bemfola^®^ and Ovaleap^®^) and one non-original biological [NOB (22)] (Primapur^®^) that had previously showed equivalence based on the primary endpoint of oocytes retrieved (23). A comparison of the three products combined versus the originator FSH medicine suggested that the rate of pregnancy outcomes was lower, although the incidence of OHSS was similar. Given that the analysis used the secondary endpoint (pregnancy outcome) from the original trials as the primary endpoint and combined three products, one of which (Primapur^®^, a NOB) did not go through the rigorous biosimilar requirements of the EMA, these results must be interpreted with caution. However, the results from the large multi-center comparative cohort study described here as well as from multi- and single center studies (20, 21) do provide further evidence that all recombinant FSH preparations, whether follitropin alfa or beta approved by the EMA and/or Australian regulatory standards, are equally effective in terms of pregnancy, live birth and cumulative live birth rates.

It is widely accepted that the ART treatment process is highly complex and subject to multiple factors that can impact clinical outcomes, such as the number of oocytes retrieved, specific laboratory procedures, embryo transfer procedure and degree of endometrial priming (24–26). Additionally, the number of ovarian follicles recruited and the endocrine environment do not affect embryo quality and ploidy status (27).

A wide range of gonadotropin preparations (urinary, purified urinary, and recombinant derived including long acting FSH) are available for ovarian stimulation. The current available evidence does not support any meaningful difference between urinary and recombinant gonadotropins regarding the rates of clinical pregnancy and live birth outcomes (28).

In the SOFIA study, the majority of women (65%) underwent fresh embryo transfer. After adjustment for embryo transfer in a univariate logistic regression analysis, there was no statistically significant difference in OHSS incidence between the two treatment groups (p = 0.233). Among women who did not undergo an embryo transfer, 13% had a freeze-all procedure. Freeze-all policy with a later frozen embryo transfer is often a preferred preventive measure to reduce late-onset OHSS risk (29). Thus, the conduct of a freeze all policy may suggest that the patient was at an increased risk for OHSS. However, the trend observed in women of reproductive age in recent years toward delaying childbearing and “social egg freezing” and the improvements achieved in the cryopreservation techniques may suggest that OHSS risk may not necessarily be the underlying factor (30, 31). Indeed, in some fertility clinics, there is an increase in practice to conduct freeze-all procedures independently of OHSS risk.

In this study, only women who had fresh embryo transfer were followed and analyzed for pregnancy outcomes. As not all women underwent fresh embryo transfer, we examined pregnancy rates in various contexts. The clinical pregnancy rates per embryo transfer were similar for both Ovaleap^®^ and Gonal-f^®^ cohorts (32% overall) as well as per oocyte retrieval (23%) and per initiated cycle (21%). Moreover, the live birth rates per pregnancy and per embryo transfer were similar between the Ovaleap^®^ and Gonal-f^®^ cohorts. Overall, spontaneous abortion rate per pregnancy was 13% and was lower than the overall loss rate of 21% reported in the literature among ART pregnancies (32). The rate of congenital anomalies in both cohorts was within the published range of approximately 2.5% reported in Europe (33).

Since the SOFIA study was a non-interventional prospective cohort study, in which patients were not randomized to treatment, the study may have been susceptible to selection bias. In addition, any unmeasured differences between the Ovaleap^®^ and Gonal-f^®^ cohorts may have contributed toward residual confounding, such as differences in clinical practice across medical centers. Selection bias and residual confounding were addressed at both design and analysis levels. At the design level, recruitment was balanced at a ratio of 1:1 for Ovaleap^®^ and Gonal-f^®^ treatment, and where possible, equal numbers of patients in each cohort from the same medical center were recruited to decrease center-based variation. Indeed, since most medical centers use both treatments in their routine practice, this variation across practices could be minimized. At the analysis level, a univariate analysis was used to adjust for risk factors or potential confounders, confirming the finding of no differences between the treatment groups. Of note, caution should be taken in interpreting results from the univariate analysis as it did not account for multiple risk factors or confounders simultaneously. As part of the statistical analysis plan for the study, a multivariate regression analysis was planned and required at least 20 patients in each subgroup to minimize the type II error rate. Due to an insufficient number of OHSS events, this pre-determined threshold was not met and the analysis was not performed.

This study may also have been susceptible to misclassification bias since exposure misclassification could have occurred if FSH treatment was switched (from Ovaleap^®^ to Gonal-f^®^ or vice versa) after study enrollment but before treatment was started. To address this potential misclassification and to account for switching, patients were classified to a cohort based on the actual treatment administered rather than the initial treatment prescribed. Such “as-treated” analysis would be more applicable for this real-world study (34). Although misclassification of OHSS is also a possibility, this would be less likely given that the study outcome was prospectively reported by physicians. To reduce misdiagnosis of OHSS, criteria for evaluation were provided and the date and severity grade were recorded. Nonetheless, any misclassification of OHSS is likely to be non-differential between the two cohorts, and thus should not affect the study results.

Given the rigorous study design, robustness of data collection and patient monitoring during the study as well as the large sample size, including patients from multiple countries and centers that were assessed for the risk of OHSS (including OHSS severity), the SOFIA study provides reliable evidence for the safety and clinical effectiveness of Ovaleap^®^ in the real-world setting.

## Conclusions

The SOFIA study examined a large cohort of patients from six European countries to assess the safety and effectiveness of Ovaleap. Findings from this study indicate that OHSS incidence proportion and severity, as well as pregnancy and live birth rates are similar between Ovaleap^®^ and Gonal-f^®^ treatments, and corroborate the safety and effectiveness of Ovaleap^®^ as a biosimilar to Gonal-f^®^ in infertile women.

## Data Availability Statement

The original contributions presented in the study are included in the article/**Supplementary Material**. Further inquiries can be directed to the corresponding author.

## Ethics Statement

The study involved human participants and was reviewed and approved by numerous ethic committees in all countries, including (but not limited to) CEIC del Hospital Clinic de Barcelona, Comité d’éthique Hospitalo-facultaire Erasme-ULB, CPP Sud-Est II, Comitato Etico Ospedale San Raffaele (CEC), East of England - Cambridgeshire and Hertfordshire Research Ethics Committee, ETHIK KOMMISSION der Ärztekammer Westfalen-Lippe und der Westfälischen Wilhelms-Universität. All participants provided their written informed consent to participate in this study.

## Author Contributions 

SK: study design, scientific oversight, and manuscript writing. RL-T: medical oversight and interpretation of results. AL and CH: interpretation of results. MD and DR: interpretation of data and results, and scientific oversight. All authors contributed to the article and approved the submitted version.

## Funding

This study was funded by Theramex and Teva Pharmaceutical Industries Ltd. (or its affiliates). The sponsors had a role in study design, scientific oversight, study management, data interpretation, manuscript writing, and decision to submit manuscript for publication.

## Disclaimer

The views expressed in this article are those of the authors and do not necessarily reflect those of Theramex or Teva Pharmaceuticals Industries Ltd. and/or its affiliates.

## Conflict of Interest

SK is an employee of Teva Pharmaceuticals Industries Ltd. RL-T is a consultant in RLT Media Consulting. RL-T was a consultant to Teva Pharmaceutical Industries Ltd. (and/or its affiliates), and then to Theramex at the time of study conduct. MD and DR are employees of the Drug Safety Research Unit (DSRU). The DSRU is an independent, academic research institution, and has received funds from Theramex for consulting on the study. CH and AL are consultants to Theramex.
