# Supplementary material for: Safety of Ovaleap® (Follitropin Alfa) in Infertile Women Undergoing Superovulation for Assisted Reproductive Technologies: A Multinational Comparative, Prospective Cohort Study
Source: Front Endocrinol (Lausanne). 2021 Mar 16;12:632674. doi: 10.3389/fendo.2021.632674 (PMC8010350; doi:10.3389/fendo.2021.632674)
Supplement: Supplementary file 1 [file Table_1.docx]

**Title:**

**Safety of Ovaleap® Follitropin Alfa in Infertile Women Undergoing Superovulation for Assisted Reproductive Technologies (SOFIA): A Multinational Comparative, Prospective Cohort Study**

**Authors:**

Sigal Kaplan^1*^, Rachel Levy-Toledano^2^, Miranda Davies^3,4^, Debabrata Roy^3,4^, Amir Lass^5^

**Affiliations and addresses:**

^1^Global Patient Safety & Pharmacovigilance, Teva Pharmaceutical Industries Ltd., Netanya, Israel

^2^ RLT Media Consulting, Boulogne Billancourt, France

^3^ Drug Safety Research Unit, Southampton, UK

^4^ School of Pharmacy and Biomedical Sciences, University of Portsmouth, Portsmouth, UK

^5^ Theramex Ltd, London, UK

**^*^Corresponding author:**

Sigal Kaplan, PhD, BPharm

E-mail address: sigal.kaplan@teva.co.il

**Supplementary Table 1.** Information collected during the routine visits for IVF cycles, SOFIA study

|  | **Enrolment** | **Visit 1** | **Visit 2** | **Pregnancy follow‑up form** |
| --- | --- | --- | --- | --- |
|  | **Start of downregulation or start of FSH treatment** | **Day of oocyte retrieval** | **Investigation of pregnancy (4 – 6 weeks after Visit 1)** | **In pregnant patients (after delivery, up to 10 – 11 months after Visit 1)** |
| Patient information and consent | x |  |  |  |
| Inclusion/exclusion criteria | x |  |  |  |
| Demographic data | x |  |  |  |
| Medical history | x |  |  |  |
| Reproductive history | x |  |  |  |
| Menstrual cycle length (days) | x |  |  |  |
| AMH level^a^ | x |  |  |  |
| Antral follicular count^a^ | x |  |  |  |
| Ovarian stimulation protocol (agonist or antagonist) |  | x |  |  |
| Study drug used (Ovaleap or Gonal-f) |  | x |  |  |
| Study drug dose administered |  | x |  |  |
| Number of follicles prior to ovulation triggering |  | x |  |  |
| Estradiol level prior to ovulation triggering |  | x |  |  |
| Ovulation triggering: product and dose |  | x |  |  |
| Oocytes retrieved (date and number) |  | x |  |  |
| Oestradiol level around time of retrieval^a^ |  | x |  |  |
| Embryo transfer (date and number) |  |  | x |  |
| Luteal phase support (product, dose and route of administration) |  |  | x |  |
| Biochemical pregnancy (beta-hCG) |  |  | x |  |
| Clinical pregnancy (assessed by ultrasound) |  |  | x |  |
| Concomitant medications | x | x^b^ | x^c^ | x |
| Adverse events |  | x | x | x^d^ |
| OHSS (following IVF cycle) |  | x | x |  |
| Pregnancy outcomes |  |  |  | x |

Abbreviations: AMH, anti-Mullerian hormone; FSH, follicle stimulating hormone; GnRH, gonadotropin-releasing hormone; hCG, human chorionic gonadotropin; IVF, in vitro fertilization; OHSS, Ovarian Hyperstimulation Syndrome.

^a^ If available

^b^ Included GnRH agonist, GnRH antagonist and product used for oocyte maturation triggering (e.g., hCG or GnRH agonist)

^c^ Included name, dose and date of administration of product used during the luteal phase support

^d^ Included abortions or ectopic pregnancies if not reported at Visit 2

**Supplementary Table 2.** Descriptive statistics for incidence of ovarian hyperstimulation syndrome for Ovaleap**®** and Gonal-f**®** cohorts by potential confounders, SOFIA study

|  | Ovaleap**®** (N=408) |  | Gonal-f**®** (N=409) |  |
| --- | --- | --- | --- | --- |
|  | Total | OHSS events | Total | OHSS events |
| **Characteristics** | n (%) | n (%) | n (%) | n (%) |
| **Age (years)** |  |  |  |  |
| <30 | 85 (21) | 6 (7) | 62 (15) | 1 (2) |
| 30 to <34 | 154 (38) | 7 (5) | 134 (33) | 4 (3) |
| ≥34 | 169 (41) | 8 (5) | 213 (52) | 8 (4) |
| **BMI (kg/m2)** |  |  |  |  |
| <18.5 | 18 (4) | 1 (6) | 25 (6) | 0 (0) |
| 18.5 to <25.0 | 257 (63) | 17 (7) | 240 (59) | 6 (3) |
| ≥25.0 | 132 (32) | 3 (2) | 142 (35) | 7 (5) |
| Missing | 1 (<1) | 0 (0) | 2 (<1) | 0 (0) |
| **PCOS** |  |  |  |  |
| No | 386 (95) | 18 (5) | 398 (97) | 11 (3) |
| Yes | 22 (5) | 3 (14) | 11 (3) | 2 (18) |
| **Menstrual cycle length** |  |  |  |  |
| < 21 Days | 3 (1) | 0 (0) | 1 (<1) | 0 (0) |
| ≥ 22 Days to ≤ 35 Days | 372 (91) | 20 (5) | 379 (93) | 11 (3) |
| > 35 Days | 33 (8) | 1 (3) | 29 (7) | 2 (7) |
| **Antral follicle count** |  |  |  |  |
| <12 | 110 (27) | 2 (2) | 128 (31) | 2 (2) |
| ≥12 | 200 (49) | 15 (8) | 179 (44) | 7 (4) |
| Missing | 98 (24) | 4 (4) | 102 (25) | 4 (4) |
| **Basal serum level of AMH** |  |  |  |  |
| <3.5 | 199 (49) | 4 (2) | 216 (53) | 3 (1) |
| ≥3.5 | 112 (27) | 15 (13) | 102 (25) | 7 (7) |
| Missing | 97 (24) | 2 (2) | 91 (22) | 3 (3) |
| **Ovarian stimulation protocol** |  |  |  |  |
| GnRH agonist | 53 (13) | 4 (8) | 52 (13) | 1 (2) |
| GnRH antagonist | 342 (84) | 17 (5) | 344 (84) | 12 (3) |
| Missing | 13 (3) | 0 (0) | 13 (3) | 0 (0) |
| **Total FSH dose** |  |  |  |  |
| <Q1 | 96 (24) | 8 (8) | 110 (27) | 4 (4) |
| Q1 to<Q3 | 210 (51) | 9 (4) | 206 (50) | 8 (4) |
| ≥Q3 | 102 (25) | 4 (4) | 93 (23) | 1 (1) |
| **FSH treatment duration** |  |  |  |  |
| <Q1 | 113 (28) | 1 (1) | 154 (38) | 0 (0) |
| Q1 to<Q3 | 186 (46) | 10 (5) | 162 (40) | 8 (5) |
| ≥Q3 | 109 (27) | 10 (9) | 93 (23) | 5 (5) |
| **FSH dose reduction** |  |  |  |  |
| No | 327 (80) | 14 (4) | 337 (82) | 6 (2) |
| Yes | 81 (20) | 7 (9) | 72 (18) | 7 (10) |
| **Oocyte maturation triggering** |  |  |  |  |
| GnRH agonist | 56 (14) | 6 (11) | 51 (13) | 7 (14) |
| hCG | 313 (77) | 15 (5) | 331 (81) | 6 (2) |
| Missing | 3 (<1) | 0 (0) | 6 (1) | 0 (0) |
| **Embryo transfer** |  |  |  |  |
| No | 136 (33) | 12 (9) | 116 (28) | 9 (8) |
| Yes | 256 (63) | 9 (4) | 274 (67) | 4 (1) |
| Missing | 16 (4) | 0 (0) | 19 (5) | 0 (0) |
| **Number of embryos transferred** |  |  |  |  |
| 1 | 143 (35) | 5 (3) | 159 (39) | 2 (1) |
| >1 | 113 (28) | 4 (4) | 115 (28) | 2 (2) |
| Missing | 152 (37) | 12 (8) | 135 (33) | 9 (7) |
| **Medications used in the luteal phase support** | |  |  |  |
| Progesterone or progestin or GnRH analogue | 267 (65) | 10 (4) | 289 (71) | 7 (2) |
| hCG | 8 (2) | 1 (13) | 10 (2) | 0 (0) |
| Missing | 133 (33) | 10 (8) | 110 (27) | 6 (5) |
| **Biochemical pregnancy^a^** |  |  |  |  |
| Negative | 172 (42) | 4 (2) | 168 (41) | 0 (0) |
| Positive | 97 (24) | 6 (6) | 112 (27) | 4 (4) |
| Missing | 139 (34) | 11 (8) | 129 (32) | 9 (7) |

Abbreviations: AMH, anti-Muellerian hormone; BMI, body mass index; FSH, follicular stimulating hormone; GnRH, gonadotropin-releasing hormone; hCG, human chorionic gonadotropin; n, number; OHSS, Ovarian Hyperstimulation Syndrome; PCOS, polycystic ovary syndrome; Q, quartile.

N was used as the denominator for calculating the percentages in the patient columns. The number of patients in the given treatment subgroup was used as the denominator for calculating the percentages in the OHSS events columns,

^a^ based on beta-hCG test

**Supplementary Table 3.** Incidence of risk factors for ovarian hyperstimulation syndrome and univariate logistic regression with adjustment for each risk factor/potential confounder, SOFIA study

| **Risk factor/Potential confounder** | **Total** | **Odds ratio** | **p-value^a^** | **Odds ratio** | **p-value^a^** |
| --- | --- | --- | --- | --- | --- |
|  |  | for confounder effect | for confounder effect | for treatment effect^b^ | for treatment effect^b^ |
|  | n (%)^c^ | (95% CI^d^) |  | (95% CI^d^) |  |
| **Age** (years) | 817 |  |  | 1.7 (0.8, 3.4) | 0.163 |
| <30 | 147 (18) | 1.1 (0.4, 2.7) | 0.751 |  |  |
| 30 - 34 | 288 (35) | 0.9 (0.4, 1.9) | 0.648 |  |  |
| ≥34 | 382 (47) | Reference |  |  |  |
| **BMI** (kg/m^2^) | 814 |  |  | 1.6 (0.8, 3.3) | 0.178 |
| <18.5 | 43 (5) | 0.5 (0.1, 3.9) | 0.594 |  |  |
| 18.5 to <25.0 | 497 (61) | Reference |  |  |  |
| ≥25.0 | 274 (34) | 0.8 (0.4, 1.7) | 0.868 |  |  |
| **PCOS** | 817 |  |  | 1.6 (0.8, 3.2) | 0.232 |
| No | 784 (96) | Reference |  |  |  |
| Yes | 33 (4) | 4.3 (1.6, 12.1) | 0.005 |  |  |
| **Menstrual cycle length** | 817 |  |  | 1.7 (0.8, 3.4) | 0.160 |
| <21 Days | 4 (<1) | <0.001(<0.001, >999.99) | 0.978 |  |  |
| ≥22 Days to ≤35 Days | 751 (92) | Reference |  |  |  |
| >35 Days | 62 (8) | 1.2 (0.3, 3.9) | 0.977 |  |  |
| **Antral follicle count** | 617 |  |  | 1.8 (0.8, 4.2) | 0.158 |
| <12 | 238 (39) | Reference |  |  |  |
| ≥12 | 379 (61) | 3.5 (1.2, 10.3) | 0.024 |  |  |
| **Basal serum level of AMH** | 629 |  |  | 1.9 (0.9, 4.2) | 0.114 |
| <3.5 | 415 (66) | Reference |  |  |  |
| ≥3.5 | 214 (34) | 6.6 (2.8, 15.6) | <0.0001 |  |  |
| **Ovarian stimulation protocol** | 791 |  |  | 1.7 (0.8, 3.4) | 0.163 |
| GnRH antagonist | 686 (87) | Reference |  |  |  |
| GnRH agonist | 105 (13) | 1.1 (0.4, 3.0) | 0.806 |  |  |
| **Total FSH dose** | 817 |  |  | 1.7 (0.8, 3.4) | 0.143 |
| <Q1 | 206 (25) | Reference |  |  |  |
| Q1 to <Q3 | 416 (51) | 0.7 (0.3, 1.4) | 0.894 |  |  |
| ≥Q3 | 195 (24) | 0.4 (0.1, 1.2) | 0.162 |  |  |
| **FSH treatment duration** | 817 |  |  | 1.4 (0.7, 3.0) | 0.312 |
| <Q1 | 267 (33) | Reference |  |  |  |
| Q1 to <Q3 | 348 (43) | 14.0 (1.9, 105.3) | 0.049 |  |  |
| ≥Q3 | 202 (25) | 20.5 (2.7, 156.7) | 0.003 |  |  |
| **FSH dose reduction** | 817 |  |  | 1.6 (0.8, 3.3) | 0.190 |
| No | 664 (81) | Reference |  |  |  |
| Yes | 153 (19) | 3.2 (1.6, 6.5) | 0.001 |  |  |
| **Oocyte maturation triggering** | 751 |  |  | 1.7 (0.8, 3.4) | 0.160 |
| GnRH agonist | 107 (14) | Reference |  |  |  |
| hCG | 644 (86) | 0.3 (0.1, 0.5) | 0.982 |  |  |
| **Embryo transfer** | 782 |  |  | 1.5 (0.8, 3.2) | 0.233 |
| No | 252 (32) | Reference |  |  |  |
| Yes | 530 (68) | 0.3 (0.1, 0.6) | <0.0001 |  |  |
| **Number of embryos transferred** | 530 |  |  | 2.5 (0.8, 8.1) | 0.140 |
| 1 | 302 (57) | Reference |  |  |  |
| >1 | 228 (43) | 1.1 (0.4, 3.4) | 0.843 |  |  |
| **Medications used in the luteal phase support** | 574 |  |  | 1.8 (0.7, 4.6) | 0.257 |
| Progesterone or progestin or GnRH analogue | 556 (97) | Reference |  |  |  |
| hCG | 18 (3) | 1.9 (0.2, 15.2) | 0.543 |  |  |
| **Biochemical pregnancy^e^** | 549 |  |  | 2.9 (0.9, 9.3) | 0.081 |
| Negative | 340 (62) | Reference |  |  |  |
| Positive | 209 (38) | 4.4 (1.4, 14.4) | 0.013 |  |  |

Abbreviations: AMH, anti-Muellerian hormone; BMI, body mass index; CI, confidence interval; FSH, follicular stimulating hormone; GnRH, gonadotrophin‑releasing hormone; hCG, human chorionic gonadotropin; n, number; PCOS, polycystic ovary syndrome; Q, quartile; UK, United Kingdom

Notes: Results were based on a series of univariate logistic regression models, including treatment and one potential confounder at a time. Odds ratio for treatment was adjusted for each of the potential confounder

^a^ p-values were based on the chi-squared test.

^b^ Treatment effect between Ovaleap® vs. Gonal-f®

^c^ Only patients with complete (non-missing) data were included in the univariate model. This number for each group was used as the denominator for calculating the percentages in that group.

^d^ 95% Wald confidence interval

^e^ based on beta-hCG test
